# A Preliminary Investigation on Smokeless Tobacco Use and Its Cognitive Effects Among Athletes

**DOI:** 10.3389/fphar.2018.00216

**Published:** 2018-03-12

**Authors:** Thomas Zandonai, Cristiano Chiamulera, Alberto Mancabelli, Danilo Falconieri, Marco Diana

**Affiliations:** ^1^Mind, Brain and Behavior Research Center, Department of Experimental Psychology, University of Granada, Granada, Spain; ^2^Neuropsychopharmacology Laboratory, Department of Diagnostic and Public Health, University of Verona, Verona, Italy; ^3^Department of Neurosciences, Biomedicine and Movement Sciences, School of Exercise and Sport Science, University of Verona, Verona, Italy; ^4^Department of Animal Biology and Ecology, University of Cagliari, Cagliari, Italy; ^5^“G.Minardi” Laboratory of Cognitive Neuroscience, Department of Chemistry and Pharmacy, University of Sassari, Sassari, Italy

**Keywords:** snus, nicotine, reinforcing effect, decision-making, winter sport environment

## Abstract

**Introduction:** Among athletes, an increasing use of nicotine via smokeless tobacco has been reported. However, there are currently unanswered questions about whether the use by athletes is due to nicotine’s addictive properties and/or to benefits in physical and cognitive performance (e.g., decision-making). In this original article we reported about, (i) snus-induced reinforcing effects among snus-user athletes (Survey) and (ii) the effects of snus on the Iowa Gambling Task (IGT) in snus-user skiers (Experimental study). IGT is an experimental neuropsychological task that has been previously used on athletes and addicts to test decision-making.

**Methods:** Survey: data were collected with the modified Cigarette Evaluation Questionnaire (mCEQ) that was administered to 61 winter sport athlete snus-users in Northern Italy. Experimental study: IGT data included: amount of money earned, number of choices from advantageous and disadvantageous decks and overall net score. Eighteen male snus-users were tested under satiety or after 12-h abstinence conditions according to a crossover design.

**Results:** Survey: the comparison between occasional vs. regular snus-users showed a statistically significant difference in satisfaction (*P* = 0.0088), calm (*P* = 0.0252), and enjoyment (*P* = 0.0001) mCEQ items suggesting a snus intake/effect relationship. Experimental study: significantly higher IGT net scores were found during the first 20 choice cards after abstinence vs. satiety conditions (*P* = 0.0024).

**Conclusion:** In the Survey, regular snus use induces greater satisfaction and psychological reward than occasional use. In the Experimental study, snus intake might produce an early and transient cognitive improvement on IGT in abstinent snus-users, presumably acting as a withdrawal relief.

## Introduction

In recent years, the literature has provided evidence supporting an increase of nicotine use in sport (Marclay and Saugy, 2010; Marclay et al., 2011; Johnston et al., 2017; Mündel, 2017) administered, mainly, via smokeless tobacco (Martinsen and Sundgot-Borgen, 2012). Snus is the smokeless tobacco product that is gaining popularity among athletes (Henninger et al., 2015). It contains and delivers quantities of nicotine comparable to those typically associate with smoking cigarettes (Foulds et al., 2003). Nicotine, is the addictive substance present in tobacco, and repeated use of nicotine induces physiological neuroadaptations (Benowitz, 2010). Snus users reported having positive expectancies about the effects (Wiium et al., 2009) and reported experiencing subjective pleasure (Caldwell et al., 2010) and exhibiting addictive behavior and withdrawal symptoms (Post et al., 2010). Snus use has a long tradition Scandinavian countries (Leon et al., 2016) particularly among males (Kvaavik et al., 2016) but not commonly reported in the other European countries (Leon et al., 2016). A recent study showed the use of snus among alpine skiers in Italy. Results showed that 74% of the athletes who practice winter sports have tried snus at least once and 50% of them continue to use it (Zandonai et al., 2016). It is unclear whether the use by athletes is due to addictive properties of nicotine or if there are real benefits to physical and cognitive performance including decision-making process.

A recent review showed that the effects of nicotine on brain circuits induce changes in decision-making processes (Naudé et al., 2015) and Mitchell (2004) demonstrated an increase in impulsive decision-making when smokers were deprived of nicotine. Decision-making is the complex process of assessing and evaluating short-term and long-term costs and benefits of competing actions (Hawthorne and Pierce, 2015) and it plays a fundamental role in most of sport activities (Kaya, 2014; Smits et al., 2014). The output of the decision-making process is determined by an interaction between impulsive or emotionally based systems, responding to immediate (potential) rewards as well as losses or threats (Bechara, 2005). An experimental tool to study decision-making is the Iowa Gambling Task (IGT) (Bechara et al., 2000; Bull et al., 2015). This test was designed to simulate real-life decisions in terms of uncertainty of outcomes and variable reward and punishment (Bechara et al., 1994; Xiao et al., 2008). Moreover, IGT was used in a sport context to test the influence of the decision-making in tactical sport behavior (Gonzaga et al., 2014) and in technical sport performance (Lage et al., 2011).

There are currently unanswered questions about the subjectively described effects and the interaction between nicotine effects and the psycho-physiological performance in training and competition. Therefore, it is important to explore the nicotine-rewarding component of snus use in the previously mentioned winter sport context. In this paper we describe the results of a survey (Survey) exploring the pattern of snus-induced reinforcing effects subjectively described by winter sport athlete snus users. Secondly, we show the effects of snus administration in a task such as IGT. Here, we present the results of the experimental study (Experimental study) on the effects of snus on decision-making among snus user skiers tested under nicotine abstinence and satiety conditions.

## Materials and Methods

### Survey

#### Participants

Sixty-one past or current snus users were recruited in Italian Dolomites area (Northern Italy), by means of local flyers and social networks. Questionnaires were administered after competitive races or during training according to their availability.

#### Questionnaire

The survey was divided into four main sections. The first section collected demographic data, the winter sport(s) practiced, the age of starting sport discipline, the competitive level and other practiced sports. Section two collected self-report data about age of first snus experience. The modified Cigarette Evaluation Questionnaire (mCEQ) (Cappelleri et al., 2007) was carried out in order to investigate the reinforcing effect of snus, which was collected in the third section. Finally, the fourth section assessed current smoking status (for details see Supplementary Material: Questionnaire).

#### Ethics

According to Italian law, in the case of the administration of anonymous questionnaires to healthy voluntary participants, the approval of an ethical committee it is not required.

#### Data Analysis

Data were analyzed by the Mann–Whitney test and unpaired *t*-test analysis (GraphPad Prism 6). Statistical significance was set at *P* < 0.05.

### Experimental Study

#### Participants

Eighteen male regular snus users were recruited through general advertisements (i.e., posters and flyers) in the metropolitan area of Verona (Italy), Trento (Italy) and on the ski slopes in the Northern Italy.

#### Ethics

The study was approved by the Ethics Committee for Clinical Trials of the University of Verona (Italy) and conformed to the 1964 Declaration of Helsinki.

#### General Design

We used a randomized crossover study design comparing the effect of snus on the IGT which is a test to evaluate the decision-making process (Bechara et al., 2000; Bull et al., 2015). IGT data include: amount of money earned, number of choices from advantageous and disadvantageous decks and overall net score. We tested participants under abstinence and satiety conditions (participants abstained from tobacco and nicotine products for a minimum of 12-h).

#### Protocol

The protocol consisted of two experimental sessions. In the first experimental session, participants arrived at the lab and they signed an informed consent form before starting the experiment. The Fagerstrom Test for Nicotine Dependence-Smokeless Tobacco (FTND-ST), a questionnaire to assess the dependence on smokeless tobacco (Ebbert et al., 2006), was assessed. Afterward, we administrated a commercial Catch White Eucalyptus Portion Snus (Swedish Match) 1.0 g – nicotine: 8 mg/portion (Zandonai et al., 2016). After 25 min of snus administration (Lunell and Curvall, 2011) participants performed IGT on the computer. We used a computerized version of the IGT with an automated method for collecting data (Pirastu et al., 2006). At the end of IGT they expelled the snus. Immediately after, 5 mL of blood were collected into pre-chilled EDTA-containing tubes. The blood was centrifuged at 3000 *g* for 10 min at 4°C and the plasma separated and stored at -20°C for nicotine and cotinine analysis that were determined by means of a high performance liquid chromatography (HPLC) technique coupled with a mass spectrometer double quadrupole detector (MS/MS) (Nakajima et al., 2000). In the second experimental session, we performed the same procedure as the first experimental session which took place at least 7 days later (wash-out time).

#### Data Analysis

Iowa Gambling Task data were analyzed using two-way ANOVA (Time and Condition). Bonferroni *post hoc* correction for multiple comparisons was applied. Abstinence vs. satiety nicotine/cotinine data was analyzed using a paired Student’s *t*-tests. All data were analyzed to confirm normality distribution using Shapiro-Wilk-W test. For the data that did not have a normally distributions a Wilcoxon signed-rank test was used. Statistical significance was set at *P* < 0.05.

## Results

### Survey

**FIGURE 1:**
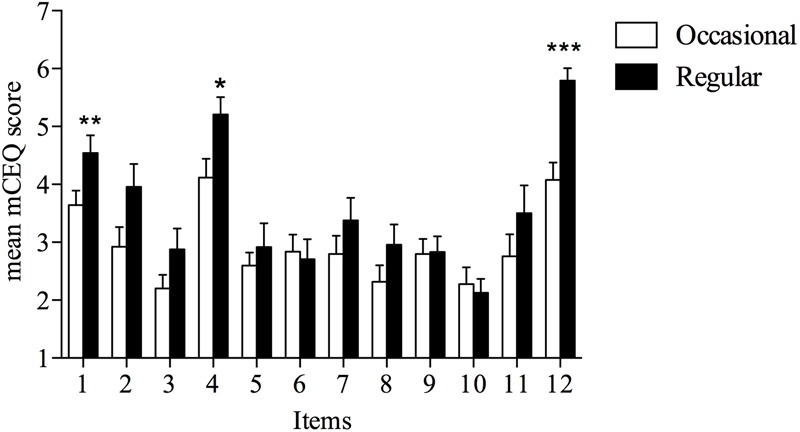
Score values (mean, SE) for each mCEQ item in occasional and regular current snus users. Bars represent occasional (white) and regular (black) snus users. Items 1 to 12 respectively represent mCEQ questions [(1) “Was using snus satisfying?,” (2) “Did snus taste good?,” (3) “Did you enjoy the sensations in your throat and chest?,” (4) “Did using snus calm you down?,” (5) “Did using snus make you feel more awake?,” (6) “Did using snus make you feel less irritable?,” (7) “Did using snus help you concentrate?,” (8) “Did using snus reduce your hunger for food?,” (9) “Did using snus make you dizzy?,” (10) “Did using snus make you nauseous?,” (11) “Did using snus immediately relieve your craving for a cigarette?,” (12) “Did you enjoy using snus?”]. ^∗^*P* < 0.05, ^∗∗^*P* < 0.01, ^∗∗∗^*P* < 0.001, unpaired Student’s *t*-test comparison between occasional current snus users versus regular current snus users for each item.

Among 61 participants, there were more males than females (51 vs. 10). The mean age was 26 years (± 5.4 SD). Fifty-five participants practiced alpine skiing, three snowboarding, two Nordic skiing and only one curling. Thirty-three participants were competitive athletes [i.e., being in possession of the medical certificate of fitness in order to participate in competitive races organized by Italian Federation of Winter Sports (F.I.S.I.)] (54% of the total), with all them beginning their winter sport discipline at 4.9 (± 3.6), years of age (± SD) (see Supplementary Table 1). At the time of the survey, 49 athletes were currently using snus (80.3% of the total). Among them 25 subjects were occasional users (one portion per week at least) and 24 were regular users (one or more cigarette a day). Thirty-two athletes were current smokers (52.5% out of the total) with 27 males (53% out of the total of males) and 5 females (50% out of the total of females). Twenty-nine participants out of 61 were current smokers (47.5%) with 24 males (47% out of the total of males) and 5 females (50% out of the total of females) as shown in Supplementary Table 2. A significant difference between occasional and regular smokers was observed for a greater number of items, that is for items 1, 2, 3, 4, 5, 6, 7, and 12 (see Supplementary Table 3), suggesting that the quantitative subjective description of smoking – induced reinforcing effect could be discriminated depending on the dose (i.e., amount of use). All mean mCEQ score values for each item were significantly different from score value = 1 (“Not at all”), thus confirming that snus use induced rewarding effects. The existence of a dose-effect relationship between snus use and reinforcing effects was tested by comparing mCEQ score values for each item in occasional vs. regular users. As shown in **Figure 1**, a significant difference in mCEQ scores between the two levels of snus use was observed only for three items: item 1 “Was using snus satisfying?” (*P* = 0.0088), item 2 “Did snus taste good?” (*P* = 0.0252) and item 12 “Did you enjoy using snus?” (*P* = 0.0001), suggesting that the existence of a dose-effect relationship was limited to few items. The average of mCEQ score for each item reported by occasional and regular current snus users is shown in Supplementary Tables 3, 4.

### Experimental Study

Participants were alpine skiers [Age = 21.4 (± 2.9), average (± SD)] and 15 out of 18 were competitive athletes. Athletes reported to use 8.1 ± 4.1 snus sachets (average ± SD) per day. The FTND-ST average value was 6.0 ± 1.7 (± SD). Only two participants were current smokers with an average of cigarettes per day of 5.5 ± 0.7 (± SD).

**FIGURE 2:**
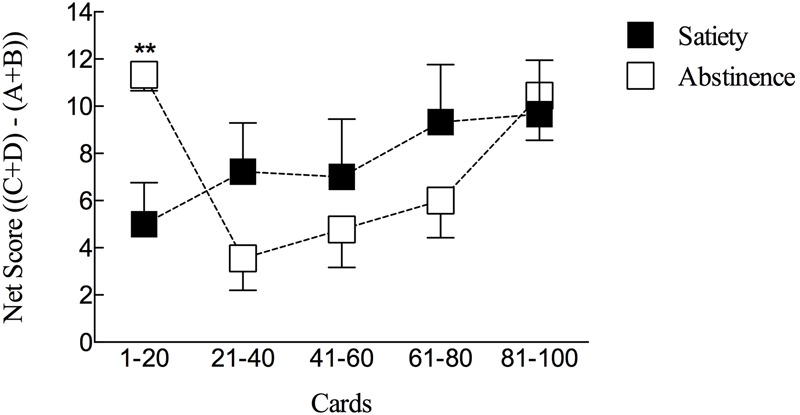
IGT net scores values for the Experimental study. Markers represent net scores values (Ordinates, mean ± SEM) for each time bin [Time 1, T1: from 1 to 21 card; Time 2, T2: from 21 to 40 card, Time 3, T3: from 41 to 60 card, Time 4, T4: from 61 to 80 and Time 5, T5: from 81 to 100 (Cards, Abscissa)] during *Experimental study* (ANOVA test). ^∗∗^*P* < 0.005.

A significant difference was observed for net scores in Time 1 (T1, from 1 to 21 card), between the abstinence and the satiety condition (*P* = 0.0024). In the Time 2 (T2, from 21 to 40 card), Time 3 (T3, from 41 to 60 card), Time 4 (T4, from 61 to 80) and Time 5 (T5, from 81 to 100), net scores values in the card blocks no significant differences were observed (**Figure 2**). No differences were observed between number of choices from advantageous decks, number of choices from disadvantageous decks and overall net score (abstinence vs. satiety). Significant difference was found in nicotine and cotinine values pre vs. post snus administration in the two conditions (*P* = 0.001; Wilcoxon test) (see Supplementary Table 5). In the IGT, the average money earned was (mean ± SEM) $ 2558 ± 233 and $ 3056 ± 293 respectively during the abstinence and satiety sessions, not significantly different (*P* = 0.1741).

## Discussion

### Survey

We confirmed anecdotal reports of snus use in winter sport athletes. This segment of the snus user population was characterized by healthy young athletes performing more than one sport, who were involved in full-time training and competitive winter sport activities (mostly Alpine and Nordic skiing) since the time of their childhood. Moreover, the survey was conducted in a European country where there is no socio-cultural tradition and marketing of snus and an increasing diffusion of this product has been recently documented (Pacifici et al., 2016; Zandonai et al., 2016). In fact, considering the lack of background knowledge in snus use in Italy, this survey, which was designed without any *ad hoc* assumption about social models, gateway hypothesis or other inductive questions. When a different sample of winter sport athletes were asked to rate the snus rewarding effects, it emerged that snus use induced effects similar to smoking tobacco, and that some of these effects – those associated with reward and satisfaction – were positively related to snus daily intake (occasional vs. regular). The comparison between the two levels of intake (occasional vs. regular snus use), showed a significant difference in satisfaction (items 1 and 12) and psychological reward (item 4) domain sub-scales. Satisfaction and psychological reward subscales are those with higher reliability among the five domains, and have been shown as moderately and positively correlated (Cappelleri et al., 2007). According to previous literature data, it could be speculated that socio-cultural and/or psychobiological factors play a role in the initiation and maintenance of this habit (Henninger et al., 2015). Italian winter sport athletes are involved in a series of winter sport competitions with a closer contact to a successful role-model (Wiium et al., 2009) such as the one offered by Northern European athletes using snus. It is also possible that the sport environment in general may facilitate the availability of snus, as reported in previous studies (Huhtala et al., 2006).

### Experimental Study

In this experimental study, snus users under abstinence conditions initially showed a significantly higher IGT score during the first time bin as compared to performance under satiety. This effect is reminiscent of those reported in abstinent smokers when tested on nicotine in cognitive tasks (Mitchell, 2004). Heishman et al. (2010) showed that nicotine-related improvement of performance is actually a relief of nicotine withdrawal cognitive impairment. Therefore, nicotine effects may help to identify the advantageous cards during the IGT as shown, for instance, in Xiao et al. (2008). We could hence hypothesize that in our study the abstinent snus users initially benefited from the nicotine effects. However, the decreasing performance in the following time bins indicates a limited duration of nicotine effects, with a trend to lower scores compared to the satiety condition. It also interesting to note that during the latter half of the IGT, inhibitory processes play an important role (Toplak et al., 2010) and they might be limited following overnight nicotine abstinence conditions (Charles-Walsh et al., 2014).

Although the published evidence (i.e., Naudé et al., 2015) seems to back up the effectiveness of nicotine on decision-making process, our data do not clarify if the hypothesis that the use of snus by athletes is due to addictive properties of nicotine. Also, until now, the literature into snus effects on exercise (Zandonai et al., 2016) and cognitive performance (i.e., Mentzoni et al., 2014 study’s did not support the hypothesis that snus users would show an attentional bias toward snus-related stimuli) has not demonstrated with clarity the real efficacy of this smokeless tobacco product. Therefore, we believe that the data of this preliminary investigation should be taken with caution. Further studies are needed that investigate snus effect on decision-making in the lab under conditions closer to the field situation, for instance under effort. This approach might allow to confirm and extend our data on snus effects on sport performance.

## Conclusion

In recent years, the interesting phenomenon of growing nicotine use in the sport environment has drawn the attention of researchers. Although nicotine is not an illegal substance and it is not considered a doping agent this is a topic to be monitored. As we showed in this article, nicotine use in regular snus users induces greater satisfaction and psychological reward than occasional use. Therefore, long-term exposure to nicotine in this segment of users might induce effects such as detrimental adaptive phenomena leading to the addiction loop rather than to a controlled recreational use. Moreover, chronic use of any psychoactive substance leads to neuroadaptive changes at a molecular and behavioral level with the risk of developing tolerance, withdrawal and dependence, rather than actual beneficial effects of sport performance (i.e., doping). Lastly, the paucity of studies on the effects of snus on decision-making suggests more research would be necessary. Particularly as far as concerns of interaction with tobacco smoking (a potential safer substitute) and on other cognitive constructs relevant in sport performance.

## Author Contributions

TZ, CC, and MD planned the experiments and wrote the draft of the manuscript. TZ and CC submitted the project to Ethics Committee for Clinical Trials of the University of Verona (Verona, Italy) for approval. TZ, CC, AM, DF, and MD carried out the experiments, analyzed the data, and revised the manuscript. All the co-authors approved the final version of the manuscript before submission.

## Conflict of Interest Statement

The authors declare that the research was conducted in the absence of any commercial or financial relationships that could be construed as a potential conflict of interest.
