# Supplementary material for: A Preliminary Investigation on Smokeless Tobacco Use and Its Cognitive Effects Among Athletes
Source: Front Pharmacol. 2018 Mar 12;9:216. doi: 10.3389/fphar.2018.00216 (PMC5857588; doi:10.3389/fphar.2018.00216)
Supplement: Supplementary file 1 [file Table_1.PDF]

Supplementary Material

A preliminary investigation on smokeless tobacco use and its cognitive effects among athletes

Thomas Zandonai*, Cristiano Chiamulera, Alberto Mancabelli, Danilo Falconieri, Marco Diana

* Correspondence
Thomas Zandonai, PhD
Mind, Brain and Behavior Research Center CIMCYC,
Dept. Experimental Psychology,
University of Granada, Spain.
Campus de Cartuja s/n,
18071, Granada, Spain.
E-mail: thomas@ugr.es

Questionnaire

First section investigated demographic data and sport information. Section two collected self-report data about age of first snus experience (At what age did you begin to use snus?). Current snus use was assessed with questions: ‘‘Use snus now?’’. Answers were ‘‘no’’, ‘‘occasionally’’ (one portion a week at least), or, ‘‘once or more time day’’ (defined as ‘regular use) (Huhtala et al., 2006). Reinforcing effects of snus was investigated with mCEQ items (third section): 1, “Was using snus satisfying?”, 2, “Did snus taste good?”, 3, “Did you enjoy the sensations in your throat and chest?”, 4, “Did using snus calm you down?”, 5, “Did using snus make you feel more awake?”, 6, “Did using snus make you feel less irritable?”, 7, “Did using snus help you concentrate?”, 8, “Did using snus reduce your hunger for food?”, 9, “Did using snus make you dizzy?”, 10, “Did using snus make you nauseous?”, 11, “Did using snus immediately relieve your craving for a cigarette?”, 12, “Did you enjoy using snus?”. These items are rated on a seven-point scale ranging from 1 (not at all) to 7 (extremely) (Caldwell et al., 2010), (Cappelleri et al., 2007). Section four investigated current smoking status. At question “Do you smoke at the present?”, answers were “no”, “occasionally” (less than one cigarette a day, defined as ‘occasional smoking) and “yes” (one or more cigarette a day, defined as ‘regular smoking’). Number of smoked cigarettes per day was also recorded. The questionnaire used to collected data was the modified Cigarette Evaluation Questionnaire (mCEQ)(Cappelleri et al., 2007) with 12 items: 1, “Was smoking satisfying?”, 2, “Did cigarettes taste good?”, 3, “Did you enjoy the sensations in your throat and chest?”, 4, “Did smoking calm you down?”, 5, “Did smoking make you feel more awake?”, 6 “Did smoking make you feel less irritable?”, 7, “Did smoking help you concentrate?”, 8, “Did smoking reduce your hunger for food?”, 9, “Did smoking make you dizzy?”, 10, “Did smoking make you nauseous?”, 11, “Did smoking immediately relieve your craving for a cigarette?”, 12, “Did you enjoy smoking?”. These items are rated on a seven-point scale ranging from 1 (not at all) to 7 (extremely).

According to Cappelleri et al. (2007), the twelve mCEQ items (both for snus and cigarette) are grouped into five domains: Satisfaction with 3 items (items 1, 2, and 12), Psychological Reward with 5 items (items 4 through 8), Aversion with 2 items (items 9 and 10), Enjoyment of Respiratory Tract Sensations (Item 3) and Craving Reduction (item 11) with one item each. Scores for each subscale were calculated as average of individual item responses. Higher scores indicated greater intensity of snus/smoking effect.

Table 1 (Survey). Demographic of participants

	Totals [%]	Male [%]	Female [%]
Participants, *N*	61 [100]	51 [83.6][a]	10 [16.4][a]
Age in years, mean (± SD)	26 (±5.4)	26.1 (±5.5)	25.9 (±5.7)
Athletes performing competitive winter sports, *N*	33 [54.0]	29 [87.8]	4 [40.0]
Alpine skiing	55 [90.2]	48 [94.1]	7 [70.0]
Snowboard	3 [4.9]	3 [5.9]	0 [0.0]
Nordic skiing	2 [3.3]	0 [0.0]	2 [20.0]
Curling	1 [1.6]	0 [0.0]	1 [10.0]
Age of starting winter sport, mean years (± SD)	4.9 (±3.6)	4.7 (±3.2)	5.6 (±5.4)
Athletes performing other exercise or sport activity, *N*	52 [85.2]	44 [86.3]	8 [80.0]

Note. [%] = percentage values of corresponding Participants column (Totals, Male or Female), except for [a] = percentage of corresponding Totals Participants value.

Table 2 (Survey). Prevalence of snus and smoking use

	Totals [%]	Male [%]	Female [%]
Participants, *N*	61	51	10
Age of first snus experience in years, mean (± SD)	20.3 (± 5.7)	19.8 (± 5.7)	22.5 (± 4.7)
No current snus users, *N*	12 [19.7]	11 [21.6]	1 [10.0]
Current snus users, *N*	49 [80.3]	40 [78.4]	9 [90.0]
Occasional	25 [41.0]	19 [37.2]	6 [60.0]
Regular	24 [39.3]	21 [41.2]	3 [30.0]
No current smokers, *N*	32 [52.5]	27 [53.0]	5 [50.0]
Current smokers, *N*	29 [47.5]	24 [47.0]	5 [50.0]
Occasional	17 [27.8]	14 [27.4]	3 [30.0]
Regular	12 [19.7]	10 [19.6]	2 [20.0]

Note. Values represent number of participants. [%] = percentage values of corresponding Participants column (Totals, Male or Female).

Table 3 (Survey). Mean (SE) score value for mCEQ items in occasional and regular current snus and smoke users.

mCEQ item	Occasional snus users (*N* 25)	Regular snus users (*N* 24)	p value	Occasional smoke users (*N* 17)	Regular smoke users (*N* 12)	p value
1	3.6 (0.2)	4.5 (0.3)	0.0088**	3.0 (0.4)	4.6 (0.4)	0.0084**
2	2.9 (0.3)	4.0 (0.4)	0.0583	2.4 (0.3)	4.8 (0.4)	0.0003***
3	2.2 (0.2)	2.9 (0.4)	0.2521	2.4 (0.3)	4.2 (0.4)	0.0045**
4	4.1 (0.3)	5.2 (0.3)	0.0252*	3.6 (0.4)	5.2 (0.3)	0.0094**
5	2.6 (0.2)	2.9 (0.4)	0.9600	2.0 (0.3)	3.5 (0.4)	0.0063**
6	2.8 (0.3)	2.7 (0.3)	0.6515	2.7 (0.3)	4.1 (0.5)	0.0177*
7	2.8 (0.3)	3.4 (0.4)	0.2968	1.9 (0.2)	3.8 (0.4)	0.0007***
8	2.3 (0.3)	3.0 (0.3)	0.1663	3.6 (0.5)	3.6 (0.5)	0.9329
9	2.8 (0.3)	2.8 (0.3)	0.8881	2.5 (0.4)	1.5 (0.2)	0.1339
10	2.3 (0.3)	2.1 (0.2)	0.8482	1.9 (0.3)	1.3 (0.1)	0.0831
11	2.8 (0.4)	3.5 (0.5)	0.2522	3.9 (0.5)	5.0 (0.6)	0.1750
12	4.1 (0.3)	5.8 (0.2)	0.0001***	3.1 (0.4)	5.0 (0.4)	0.0012**

Note. *P < 0.05, **P < 0.01, ***P < 0.001, unpaired Student's t-test comparison between occasional current users versus regular current users for each item.

Table 4 (Survey). Mean score (SE) mCEQ score values pooled into five domains for current snus users and smokers.

Domain	Current snus user		Current smoke users	
	Occasional	Regular	Occasional	Regular
Satisfaction	3.5 (0.3)	4.8 (0.3)	2.8 (0.2)	4.8 (0.1)
Psychological reward	2.9 (0.3)	3.4 (0.5)	2.8 (0.4)	4 (0.3)
Enjoyment of respiratory tract sensations	2.2 (0.2)	2.9 (0.4)	2.4 (0.3)	4.2 (0.4)
Craving reduction	2.8 (0.4)	3.5 (0.5)	3.9 (0.5)	5 (0.6)
Aversion	2.5 (0.3)	2.5 (0.4)	2.2 (0.3)	1.4 (0.1)

Note. Satisfaction = items 1, 2, and 12; Psychological Reward = items 4, 5, 6, 7 and 8; Enjoyment of Respiratory Tract Sensation = item 3; Craving Reduction = item 11; Aversion = items 9 and 10.

Iowa Gambling Task

Four decks of cards labelled A, B, C and D were displayed on the computer screen. The backs of the cards all look the same as real decks of cards. The participant started the task with a sum of make-believe money in his or her account ($2,000), represented by a green bar that changes in length as the participant ‘‘wins’’ or ‘‘loses’’ money during the task. The participant was required to select one card at a time from one of the four decks. When the participant selected a card, a message was displayed on the screen indicating the amount of money the participant won or lost. The computer controlled the pre-programmed schedules of gain and loss. Turning each card gave an immediate reward of $100 in Decks A and B and $50 in Decks C and D. As the game progressed, there were also unpredictable losses among the card selection. Total losses amounted to $1,250 in every 10 cards in Decks A and B compared to $250 in Decks C and D. Decks A and B were equivalent in terms of overall net loss, and Decks C and D were equivalent in terms of overall net gain over the course of the trials. The difference was that in Decks A and C, the punishment was more frequent but of smaller magnitude, whereas in Decks B and D, the punishment was less frequent but of higher magnitude. Thus, Decks A and B were disadvantageous because they yielded high immediate gain but a greater loss in the long run (i.e., net loss of $250 for every 10 cards), and Decks C and D were advantageous in that they yielded lower immediate gain but a smaller loss in the long run (i.e., net gain of $250 for every 10 cards). In this study, an overall net score of the IGT was calculated by subtracting the total number of selections from disadvantageous decks (A and B) from total number selections from advantageous decks (C and D). We divided all 100 cards in five block cards called Time 1 (T1, from 1 to 21 card), Time 2 (T2, from 21 to 40 card), Time 3 (T3, from 41 to 60 card), Time 4 (T4, from 61 to 80) and Time 5 (T5, from 81 to 100) respectively and we calculated the net scores values ((C + D) – (A + B)) for each blocks cards (Balodis et al., 2006).

Table 5 (Experimental Study). Plasma nicotine and cotinine concentration under abstinence and satiety conditions.

	**Nicotine (ng/ml)**			**Cotinine (ng/ml)**		
	Pre	Post	P values	Pre	Post	P values
**AB**	0.8 ± 1.5	8.3 ± 3.7	< 0,0001***	59.9 ± 96.9	133.7 ± 105.5	0.0290*
**SA**	9.4 ± 10.6	8.3 ± 7.4	0.5226	238.1 ± 249.8	158.2 ± 204.7	0.0585

AB: Abstinence Condition, SA: Satiety Condition, (means ± SD) *P < 0.05, ***P < 0.001, paired Student's t-test comparison between pre and post nicotine administration by snus.
